# Status of common sexually transmitted infection in population referred for colposcopy and correlation with human papillomavirus infection

**DOI:** 10.1186/s12905-023-02693-6

**Published:** 2023-11-08

**Authors:** Disi A, Jiayue Li, Dai Zhang, Bingbing Xiao, Hui Bi

**Affiliations:** 1 Department of Obstetrics and Gynecology, Peking University First Hospital, Beijing, 100034 China; 2 School of Medicine, University College Dublin, Dublin, Ireland

**Keywords:** Human papillomavirus, Sexually transmitted infection, Colposcopy, Cervical intraepithelial neoplasia, *Chlamydia trachomatis*, *Ureaplasma parvum*

## Abstract

**Background:**

To investigate the prevalence of common sexually transmitted infections (STIs) and the association of STI/human papillomavirus co-infection in young and middle-aged women with previous abnormal cervical findings referred for colposcopy.

**Methods:**

719 cervical-swab cytobrush specimens were obtained from women aged ≤ 50 years who were referred for colposcopy at Peking University First Hospital due to previous abnormal cervical findings. HPV 21 typing and a panel of pathogenic STIs were tested for using the 21 HPV GenoArray Diagnostic Kit (HBGA-21PKG; HybriBio, Ltd., Chaozhou, China) and a nucleic acid STI detection kit (HybriBio Ltd. Guangzhou, China), after which colposcopy with multipoint positioning biopsy was performed.

**Results:**

The overall prevalence of STIs among HPV positive women with previous abnormal cervical cancer screening results was 63.7% (458/719), with *Ureaplasma parvum* serovar 3, *Ureaplasma parvum* serovar 6 and herpes simplex virus type 2 having significantly higher prevalence among high-risk HPV positive patients (19.3%, Χ^2^ = 5.725, P = 0.018; 21.5%, Χ^2^ = 4.439, P = 0.035; 5.7%, Χ^2^ = 4.184, P = 0.048). Among patients positive for the high-risk human papillomavirus, the prevalence of *Neisseria gonorrhoeae* infection in human papillomavirus 16/18 positive patients was significantly higher than that in other patients (2.5%, Χ^2^ = 4.675; P = 0.043). Histopathologically, *Chlamydia trachomatis* infection was more frequently detected in lower than or equal to low-grade squamous intraepithelial lesion infection status (13.0%, Χ^2^ = 3.368; P = 0.041).

**Conclusions:**

The high prevalence of HPV coinfection with other sexually transmitted pathogens, particularly *Ureaplasma parvum* serovar 3, *Ureaplasma parvum* serovar 6, and herpes simplex virus type 2, calls for routine STI screening and effective STI prevention and management in patients with abnormal cervical cancer screening results.

**Supplementary Information:**

The online version contains supplementary material available at 10.1186/s12905-023-02693-6.

## Background

Cervical cancer (CC) is a serious threat to women’s health worldwide, with 569, 000 new cases and 311, 000 deaths worldwide in 2018, cervical cancer is the fourth most common cancer in women worldwide [1]. More than 200 HPV subtypes have been identified to infect humans, of which 14 subtypes (16, 18, 31, 33, 35, 39, 45, 51, 52, 56, 58, 59, 66 and 68 subtypes) are high-risk HPV (HR-HPV) [2]. HPV infection is associated with many reproductive health complications, including cervical, anal, and oropharyngeal cancers [3, 4].

Sexually transmitted infections (STIs) are the most common infectious diseases worldwide, with an estimate of 1 million cases reported every day according to a 2016 World Health Organization study [5]. STIs are also associated with reproductive health complications including pelvic inflammatory disease, infertility, ectopic pregnancy, miscarriage, neonatal death, neonatal infectious diseases, and even cardiovascular and neurological diseases [6].

More and more recent studies have shed light on the crucial role of STIs in the development persistent HPV infections, by facilitating the entry of HR-HPVs and diminishing the host’s defend against the HPV infection, which could lead to the development of cervical cancer [7]. However, there is limited data on the association of STIs and HPV infection in Chinese population specifically. Furthermore, women with an abnormal cervical cancer screening results are referred for coloscopy to detect precancer according to current guideline, and exploring the possible association between STIs and HPV infection could provide recommendations for a more comprehensive examination and treatment of women who are already at high risk of developing cervical cancer and other complications with existing abnormal cervical cancer screening results.

## Methods

### Study design

This cross-sectional study was reviewed and approved by The Ethics Committee of Peking University First Hospital (2021KY062).

In total, 719 samples were collected from women who attended Peking University First Hospital for colposcopy referral from June 2021 to November 2021 in Beijing, China. Women with a colposcopy referral were chosen as the study population as a colposcopy referral indicates previous abnormal cervical findings, which put them at a higher risk of developing cervical cancer and other complications.

The inclusion criteria were women aged 19 to 50 years who attended Peking University First Hospital for colposcopy referral. The exclusion criteria were women who (1) were menopausal or menstruating, (2) were virgins, pregnant, or within 8 weeks postpartum, (3) used vaginal medication or vaginal irrigation within 3 days of sampling, (4) had vaginal bleeding, (5) had a history of genital tract tumors, (6) had been recently treated for HPV infection or STI, 6) had hysterectomy, cervical surgery, pelvic radiotherapy, or cervical ablation/resection within the last 12 months, and (7) used antibiotics or probiotics within the past month.

All study participant had had previous abnormal cervical findings either at Peking University First Hospital or elsewhere. Upon enrollment, cervical samples were collected for both HPV 21 genotyping and STI detection, after which colposcopy and biopsy were performed. The samples were collected by physicians who received standardized colposcopy training, and they were placed in nucleic acid detection preservation solution and stored at 4 °C for HPV 21 typing and STI detection.

### Detection of HPV infection

The 21 HPV GenoArray Diagnostic Kit (HBGA-21PKG; HybriBio, Ltd., Chaozhou, China), an HPV HybriMax™ medical nucleic acid molecular rapid hybridization instrument was used for HPV identification and genotyping. The kit detects 21 HPV genotypes, including 14 high-risk genotypes (HPV16, -18, -31, -33, -35, -39, -45, -51, -52, -56, -58, -59, -66, -68), 1 suspected high-risk genotype (HPV53) and 6 low-risk genotypes (HPV6, -11, -42, -43, -44 and -CP8304) [8].

### Detection of sexually transmitted pathogens

A nucleic acid detection kit (HybriBio Ltd. Guangzhou, China) was used for the identification of sexually transmitted pathogens including *Chlamydia trachomatis* (*C. trachomatis*), *Neisseria gonorrhoeae* (*N. gonorrhoeae*), herpes simplex virus type 2 (HSV-2), and *Mycoplasma* subtypes, including *Mycoplasma hominis* (*M. hominis*), *Mycoplasma genitalium* (*M. genitalium*), *Ureaplasma urealyticum* (*U. urealyticum*) and *Ureaplasma parvum* (*U. parvum*, serovar 1, 3, 6 and 14). This test employs a patented flow-through Hybridization technique using real-time fluorescent PCR fluorescent probe method to detect STI microorganisms,and it was carried out following the manufacturer’s instructions [9].

### Colposcopy

Participants had all attended a cervical cancer screening either at Peking University First Hospital or elsewhere, received an abnormal screening test result, and were referred to Peking University First Hospital for colposcopy to detect precancer [10].

All colposcopy examinations involved standardized evaluations of the cervix by a trained clinician using magnification after application of 3–5% acetic acid. Transformation zones and lesions were assessed and classified according to the 2011 IFCPC (International Federation for Cervical Pathology and Colposcopy) guideline [11]. Multipoint localization biopsy was performed for any abnormal area observed by colposcopy, and Endocervical Curettage (ECC) was performed if no abnormality was observed, or abnormality was inconsistent with the screening results. All specimens were sent for pathological examination. Cervical intraepithelial lesions were classified according to the WHO 2020 two-grade classification of high-grade squamous intraepithelial lesion (HSIL) and low-grade squamous intraepithelial lesion (LSIL) [12, 13].

### Statistical analysis

Data were analyzed using the SPSS 28.0 software (IBM, Armonk, NY, USA). Single infection was defined as testing positive for one microorganism, while multiple infections was defined as testing positive for two or more microorganisms. Frequency data were summarized with percentage of cases. Chi-square and Fisher’s exact probability tests were conducted to test for associations. A 2-sided P value less than 0.05 was considered statistically significant. In accordance with the journal’s guidelines, we will provide our data for independent analysis by a selected team by the Editorial Team for the purposes of additional data analysis or for the reproducibility of this study in other centers upon requested.

### Patient and public involvement

Patients and members of the public were not involved in the design of this study.

## Results

### STIs in the HR-HPV positive population referred for colposcopy

The ages of the 719 subjects included in this study ranged from 19 to 50 years, with a mean age of 35.36 ± 0.258 years (ranging from 19 to 50 years). The specific information of demographic and clinical data analyzed by HPV status was shown in Supplementary Table 1. The positive rate of HPV infection was 87.6% (630/719), of which 97.6% (615/630) were HR-HPV. The total positivity rate of detected STI pathogens was 63.7% (458/719), mainly single pathogen infection (69.0%, 316/458).

The patients were divided into HPV negative group, HPV single infection group and HPV multiple infection group. Among the three groups, significant difference was observed in the negativity rate, single positivity rate and multiple positivity rate of detected STI pathogens (Χ^2^ = 24.406, P = 0.000), as shown in Supplementary Table 2.

**Fig. 1:**
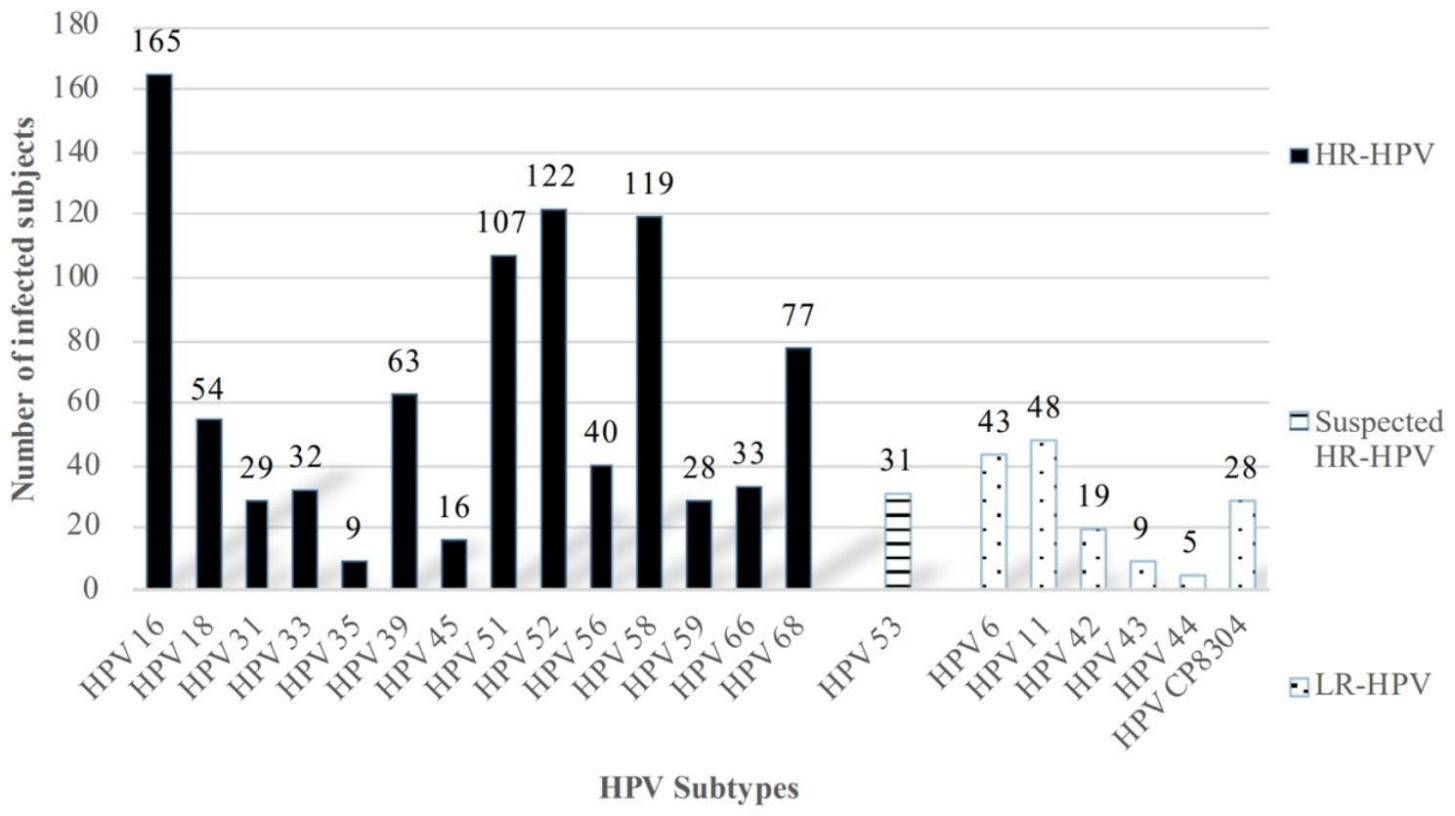
Prevalence of subjects infected by different HPV types HPV, Human Papillomavirus. Solid-color bars: High-risk HPV (HR-HPV); Striped bar: Suspected HR-HPV; Speckled bars: Low-risk HPV (LR-HPV).

The 15 HR-HPV subtypes tested were ranked and the top three were HPV16 (26.8%, 165/615), HPV52 (19.8%, 122/615) and HPV58 (19.3%, 119/615) (Fig. 1).

**Fig. 2:**
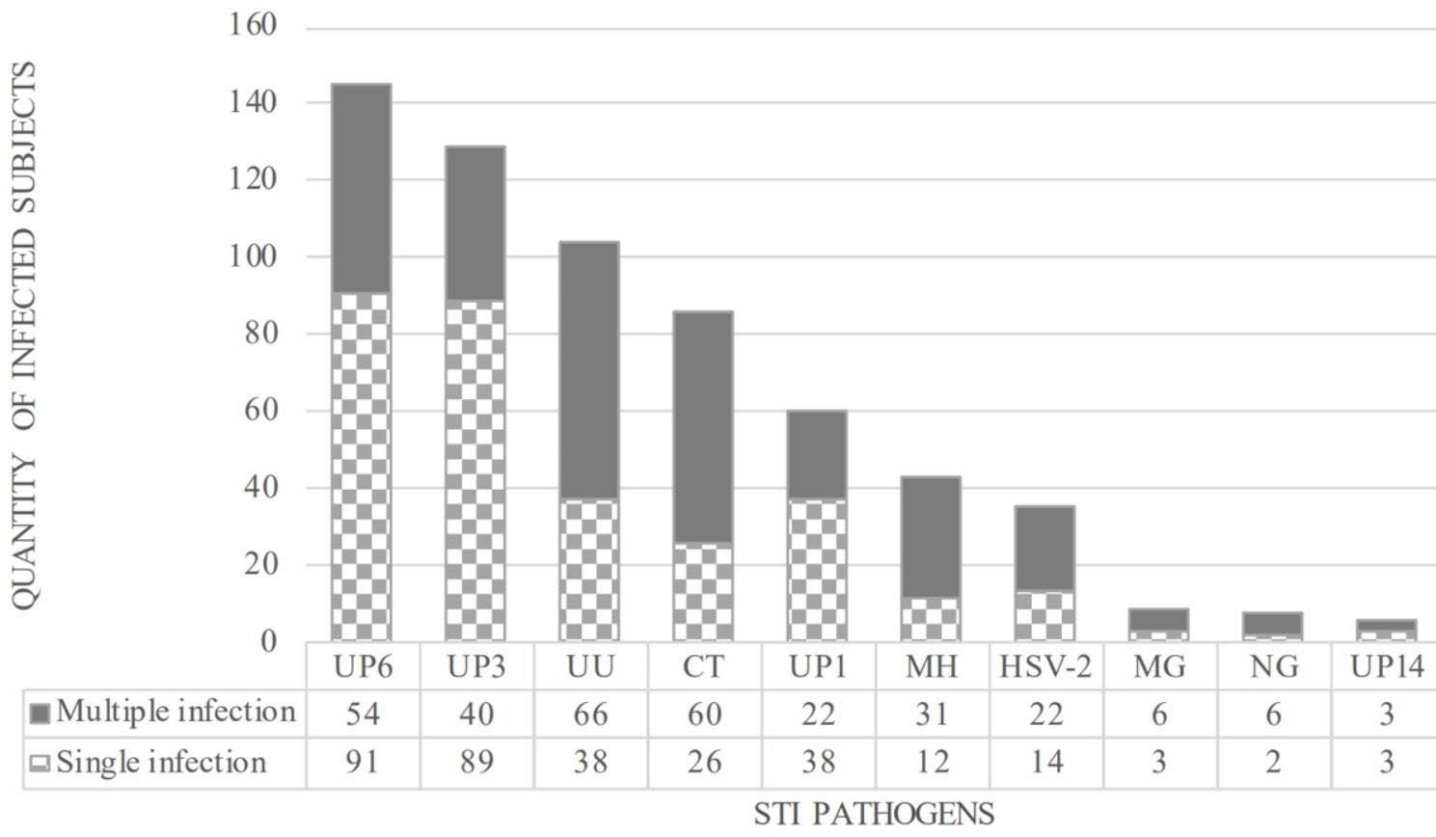
Infection status of different STI pathogens in the total population (N = 719) Solid-color bars: Multiple infection. Speckled bars: Single infection. STI, Sexually Transmitted Infection; CT, *Chlamydia Trachomatis*; UU, *Ureaplasma urealyticum*; MH, *Mycoplasma hominis*; MG, *Mycoplasma genitalium*; UP, *Ureaplasma parvum*; NG, *Neisseria Gonorrhoeae*; HSV-2, herpes simplex virus 2

The total positive rate of the detected STI pathogens in the HR-HPV positive population was 65.5% (403/615), mainly sustained by single infection (68.7%, 277/403). The three most prevalent STI pathogens in the total population were *U. parvum* serovar 6 (20.2%,145/719), *U. parvum* serovar 3 (17.9%, 129/719) and *U. urealyticum* (14.5%, 104/719) (Fig. 2).

There were significant differences in the single positive rate and multiple positive rates of detected STI pathogens between HR-HPV positive and negative groups (Χ^2^ = 6.242, P = 0.044). Compared with HR-HPV negative group, *U. parvum* serovar 3, *U. parvum* serovar 6 and HSV-2 infection rates were significantly higher in HR-HPV positive group (Χ^2^ = 5.725, P = 0.018; Χ^2^ = 4.439, P = 0.035; Χ^2^ = 4.184, P = 0.048), as shown in Table 1.

**Table 1:** Comparison of infection rate of detected STI pathogens under different HR-HPV infection status

STIs	Total / N	HR-HPV Positive N = 615	HR-HPV negative N = 104	Х^2^	P
*C. trachomatis*	86	75 (12.2%)	11 (10.6%)	0.221	0.745
*U. urealyticum*	104	85 (13.8%)	19 (18.3%)	1.423	0.230
*M. hominis*	43	37 (6.0%)	6 (5.8%)	0.010	1.000^*^
*M. genitalium*	9	9 (1.5%)	0 (0.0%)	1.541	0.371
*U. parvum* serovar 1	60	49 (8.0%)	11 (10.6%)	0.792	0.343
*U. parvum* serovar 3	129	119 (19.3%)	10 (9.6%)	5.725	0.018
*U. parvum* serovar 6	145	132 (21.5%)	13 (12.5%)	4.439	0.035
*U. parvum* serovar 14	5	5 (0.8%)	1 (1.0%)	0.024	1.000^*^
*N. gonorrhoeae*	8	7 (1.1%)	1 (1.0%)	0.025	1.000^*^
HSV-2	36	35 (5.7%)	1 (1.0%)	4.184	0.048
Single infection	318	277 (45.0%)	39 (37.5%)	6.242	0.044
Multiple infection	142	126 (20.5%)	16 (15.4%)		

* Fisher’s exact test. Multiple infections double counting

HR-HPV, high-risk human papillomavirus; STIs, sexually transmitted infections; *C. trachomatis*, *Chlamydia Trachomatis*; *U. urealyticum*, *Ureaplasma urealyticum*; *M. hominis*, *Mycoplasma hominis*; *M. genitalium*, *Mycoplasma genitalium*; *U. parvum*, *Ureaplasma parvum*; *N. gonorrhoeae*, *Neisseria Gonorrhoeae*; HSV-2, herpes simplex virus 2

### STIs of populations infected with different HR-HPV subtypes

In the HR-HPV positive group, positivity rates of the detected pathogens between patients with or without HPV16/18 infection were compared in Supplementary Table 3. No significant differences were observed in the negativity rates, single pathogen positivity rates or multiple pathogen positivity rates of the detected STIs between the two groups (Χ^2^ = 1.502, P = 0.472). *N. gonorrhoeae* infection rate was significantly higher in patients with HPV 16/18 infection (Χ^2^ = 4.675, P = 0.043).

### STIs among subjects with different histopathological results

Among 719 cases evaluated by colposcopy, 593 (82.5%, 593/719) and 126 cases (17.5%, 126/719) were diagnosed as lower than or equal to LSIL (≤ LSIL) and higher than or equal to HSIL (≥ HSIL) respectively by histopathology. The positive rates of the detected pathogens between cases diagnosed by histopathology as ≤ LSIL and ≥ HSIL were compared in Table 2. No significant difference was observed among patients in the groups who were negative for the detected STI pathogens, those infected with single pathogens and those infected with multiple pathogens (X^2^ = 1.089, P = 0.580). The *C. trachomatis* infection rate was significantly higher in the cases diagnosed by histopathology as ≤ LSIL (X^2^ = 3.368, P = 0.041).

**Table 2:** Comparison of positive rates of detected STI pathogens among different histopathological results

STIs	Total /N	≤LSIL N = 593	≥HSIL N = 126	Х^2^	P
*C. trachomatis*	86	77 (13.0%)	9 (7.1%)	3.368	0.041
*U. urealyticum*	104	81 (13.7%)	23 (18.3%)	1.733	0.118
*M. hominis*	43	34 (5.7%)	9 (7.1%)	0.3674	0.536
*M. genitalium*	9	7 (1.2%)	2 (1.6%)	0.139	0.487
*U. parvum* serovar 1	60	47 (7.9%)	13 (10.3%)	0.777	0.577
*U. parvum* serovar 3	129	110 (18.5%)	19 (15.1%)	0.850	0.443
*U. parvum* serovar 6	145	113 (19.1%)	32 (25.4%)	2.595	0.113
*U. parvum* serovar 14	6	4 (0.7%)	2 (1.6%)	1.046	0.284
* N. gonorrhoeae*	8	8 (1.3%)	0 (0.0%)	1.719	0.363
HSV-2	36	32 (5.4%)	4 (3.2%)	1.078	0.374
Single infection	316	264 (44.5%)	52 (41.3%)	1.089	0.580
Multiple infection	142	113 (19.1%)	29 (23.0%)		

Multiple infections double counting

LSIL, low-grade squamous intraepithelial lesion; HSIL, high-grade squamous intraepithelial lesion; STIs, sexually transmitted infections; *C. trachomatis*, *Chlamydia Trachomatis*; *U. urealyticum*, *Ureaplasma urealyticum*; *M. hominis*, *Mycoplasma hominis*; *M. genitalium*, *Mycoplasma genitalium*; *U. parvum*, *Ureaplasma parvum*; *N. gonorrhoeae*, *Neisseria Gonorrhoeae*; HSV-2, herpes simplex virus 2

## Discussion

This study investigated the prevalence of sexually transmitted pathogens and co-infection with HPV among women with previous abnormal cervical findings in Beijing, China. The study demonstrated a high overall STI prevalence among patients with a colposcopy referral (63.7%), which is similar to the positivity rate of 7 STI pathogens (49.2%) in the colposcopy referral population studied by Martinelli et al. [14] and the positivity rate of detected STI pathogens (63.4%) in 320 high-risk HPV-positive patients studied by Lei Wang et al. [15]. According to WHO data, 40% of Chinese women experience different degrees of genital tract infection, and more than 55% of gynecological outpatients experience genital tract infection [16].

The infection status of *C. trachomatis* needs much attention. In our study, the *C. trachomatis* infection rate of young and middle-aged women referred to colposcopy was 12.0%, which is much higher than the global average incidence rate (3.4%) [17] and the infection rate of screened women in Beijing (2.2%) [18]. These rates were also higher than the incidence rate of 5% in the HR-HPV positive population in the study by Lei Wang et al. [15] and were consistent with the results of Martinelli et al., who collected cervical specimens with abnormal cytology [19]. *C. trachomatis* has been found to be associated with cervical HSIL in cases of HPV coinfection in many existing studies, while the difference in the results of this study may be due to the small study population. A meta-analysis of 22 studies found that *C. trachomatis* increased the risk of cervical cancer development in both prospective and retrospective studies (P < 0.001; P < 0.001) [20]. Regarding the mechanism, it was found that *C. trachomatis* mainly parasitizes epithelial cells of the cervix, causes long-term chronic recurrent infections and urinary reproductive system inflammation, induces local immune induction and secretion of mediators and enhances reactive oxygen species (ROS) as well as free radical generation, which could lead to a host mucosal barrier and cell-mediated immune injury [21, 22]. Besides, coinfection of HPV and *C. trachomatis* was found to create an environment for cellular transformation, activates an innate immune response and triggers epithelial-mesenchymal transition [23]. Other studies have also reported that people with *C. trachomatis* have an increased risk of HPV infection [24, 25].

The correlation between *U. urealyticum* and HPV infection has not yet been clarified. Kim et al. [26] enrolled 264 asymptomatic patients and differentiated colonization and infection with 10^4^ CCU/mL as the threshold. They found that *U. urealyticum* of high-density colonization (> 10^4^ CCU/mL) was significantly correlated with HPV infection (P = 0.014). A study of 1218 Chinese women reported no significant association between *U. urealyticum* infection and HR-HPV infection (P = 0.619), although the prevalence of *U. urealyticum* in healthy women was 35.5% [18]. Another study including 2215 patients suggested that *U. urealyticum* infection was correlated not only with HR-HPV infection but also with Cervical Intraepithelial Neoplasia (CIN) (P < 0.05) [27].

At present, most studies suggested that *M. hominis*, *M. genitalium* may correlated with HPV infections. A meta-analysis of 22 studies suggested that *M. genitalium* was significantly associated with an increased risk of HR-HPV infection and that *M. hominis* was significantly associated with an increased risk of CIN [28]. Epidemiological and nucleic acid analysis of 1160 Tanzanian women by Klein et al. [29] also showed a significant association between persistent *M. hominis* infection and persistent HR-HPV infection.

Few studies have been published on *U. parvum* and HPV infection. Healthy people have been reported to possibly carry *U. parvum* as well. Noma et al. [30] reported a 5-fold increased risk of LSIL in women who were positive for *U. parvum* (P = 0.02). However, few relevant studies on the specific 4 serotypes of *U. parvum* have been published. Zanotta et al. [31] found that, of all *U. parvum* serotypes, *U. parvum* serovar 3 was the main colonizing bacterium in the urogenital tract and was significantly associated with coinfection with other STI pathogens (P < 0.01), as 24.3% of asymptomatic HR-HPV patients in the study were coinfected with *U. parvum* serovar 3. Another retrospective study enrolled 668 patients and found that *U. parvum* serovar 3, *U. parvum* serovar 6 and *M. genitalium* could promote persistent HR-HPV infection and accelerate CIN development, thus aggravating HPV-mediated cervical lesions [32]. According to the study by Lei Wang et al., *U. urealyticum* and *U. parvum* serovar 1 were associated with HPV negativity, while *U. parvum* serovar 14 increased the risk of HSIL and invasive cancer [15].

Regarding the correlation between *N. gonorrhoeae*, HSV-2, or HPV infection and CC, no unified conclusions have been established. A cross-sectional study of 9090 Chinese women found that *N. gonorrhoeae* infection in the genital area was associated with an increased risk of CIN after adjustment for HR-HPV-positive status [33], but Paula et al. [34] found no significant correlation between *N. gonorrhoeae* infection and cervical lesions. As the cause of most recurrent cases of genital herpes, HSV-2 has been found to induce cervical tissue carcinogenesis in animal models, which may be related to DNA transfection that can interfere with the cell cycle, lead to the accumulation of abnormal mutations and destroy the stability of the host genome [35]. When available continuous cross-sectional data from the National Health and Nutrition Examination Survey (NHANES) from 1999 to 2014 were examined, after further adjustment for HR-HPV as a confounding factor, HSV-2 was associated with CC. The serum status of HSV-2 can be an independent predictor of CC [36].

Furthermore, the progress of STI research in the field of vaginal self-sampling is also noteworthy. Though all the patients in this study were sampled in the hospital, more convenient and flexible approach for STI testing sampling is needed in real life. As early as 2007, Lippman et al. [37] found that home-based self-collection and self-testing of STI was acceptable, feasible, and even resulted in a slightly higher response rate than clinic-based screening. Sechi et al. [38] also found that three different vaginal self-collection devices had a similar agreement for HR-HPV detection as compared to cervical samples. Some other similar studies in recent years also proved the value of vaginal self-sampling for STI testing [39, 40], which suggested that integrating the vaginal self-sampling into the clinical workflow and scientific research might be a more efficient approach [41].

The main limitations of this study were the cross-sectional observational design and the relatively small sample size. In addition, the quantity of study population with multiple STI infections was relatively small, affecting further refinement of the analysis.

The STI infection rate in the population undergoing colposcopy in this study was significantly higher than the general prevalence of STI in Chinese women, which indicates that it is necessary to improve the screening of STI pathogens in the population undergoing colposcopy, especially in patients with a positive HPV test. Screening for STI pathogens is recommended in HR-HPV-positive individuals of reproductive age referred for colposcopy. Among them, the screening for *C. trachomatis* in individuals who undergo colposcopy and actively administer treatment needs more attention. Besides, the detailed clinical classification of *Mycoplasma* would be helpful to determine its role in the occurrence and development of HR-HPV-related diseases.

## Conclusions

In conclusion, attention should be given to the screening and post-screening management of STI pathogens in young and middle-aged women referred for colposcopy. The positive expression of *U. parvum* serovar 3, *U. parvum* serovar 6 and HSV-2 might be related to HR-HPV infection. The *C. trachomatis* infection rate was high in young and middle-aged patients with colposcopy referral indications. Further longitudinal studies are needed to investigate the role of STI as a cofactor in HR-HPV-induced cervical lesions.

### Electronic supplementary material

Below is the link to the electronic supplementary material.

Supplementary Material 1

## Data Availability

All data generated or analyzed during this study are included in this published article [and its supplementary information files].

## Abbreviations

CC: Cervical cancer
CIN: Cervical Intraepithelial Neoplasia
*C. trachomatis*: *Chlamydia trachomatis*
ECC: EndoCervical Curettage
HSIL: High-grade squamous intraepithelial lesion
HPV: Human papillomavirus
HR-HPV: High-risk HPV
HSV-2: Herpes simplex virus type 2
IFCPC: International Federation for Cervical Pathology and Colposcopy
LSIL: Low-grade squamous intraepithelial lesion
*M. genitalium*: *Mycoplasma genitalium*
*M. hominis*: *Mycoplasma hominis*
*N. gonorrhoeae*: *Neisseria gonorrhoeae*
NHANES: National Health and Nutrition Examination Survey
ROS: Reactive oxygen species
STI: Sexually transmitted infection
*U. parvum*: *Ureaplasma parvum*
*U. urealyticum*: *Ureaplasma urealyticum*
