# Supplementary material for: Status of common sexually transmitted infection in population referred for colposcopy and correlation with human papillomavirus infection
Source: BMC Womens Health. 2023 Nov 8;23:579. doi: 10.1186/s12905-023-02693-6 (PMC10634156; doi:10.1186/s12905-023-02693-6)
Supplement: Supplementary file 1 — Supplementary Material 1 [file 12905_2023_2693_MOESM1_ESM.pdf]

Supplementary Table 1 Demographic and clinical data analysed by HPV status

	HPV Negative<br>N (%)	HPV Positive<br>N (%)	Total<br>N (%)	P
**Age**				
19-26	13 (14.6)	64 (10.2)	77 (10.7)	
27-34	21 (23.6)	237 (34.6)	258 (35.9)	0.002
35-42	46 (51.7)	218 (34.6)	264 (36.7)	
43-50	9 (10.1)	111 (17.6)	120 (16.7)	
**Vaginal pH**				
3.8-4.5	77 (86.5)	535 (84.9)	612 (85.1)	0.692
>4.5	12 (13.5)	95 (15.1)	107 (14.9)	
**Bacterial vaginosis**				
Negative	73 (82.0)	510 (81.0)	583 (81.1)	0.716
Intermediate	11 (12.4)	70 (11.1)	81 (11.3)	
Positive	5 (5.6)	50 (7.9)	55 (7.6)	
**Aerobic Vaginitis**				
Negative	78 (87.6)	549 (87.1)	627 (87.2)	0.895
Positive	11 (12.4)	81 (12.9)	92 (12.8)	
**Vaginal Trichomoniasis**				1.000*
Negative	89 (100.0)	629 (99.8)	718 (99.9)	

Positive	0 (0.0)	1 (0.2)	1 (0.1)	
**Vulvovaginal Candidiasis**				
Negative	89 (100.0)	609 (96.7)	698 (97.1)	0.095*
Positive	0 (0.0)	21 (3.3)	21 (2.9)	

* Fisher's exact test.

HPV, human papillomavirus.

Supplementary Table 2 Comparison of negative rate, single positive rate and multiple positive rates of 10 STI pathogens in different HPV infection types

HPV infection type	STI infection type			
	Negative N (%)	Single infection N (%)	Multiple infection N (%)	Total N (%)
Negative	44 (16.9)	31 (9.8)	14 (9.9)	89 (12.4)
Single HPV infection	135 (51.7)	161 (50.9)	51 (35.9)	347 (48.3)
Multiple HPV infections	82 (31.4)	124 (39.2)	77 (54.2)	283 (39.4)
Total	261 (36.4)	316 (43.9)	142 (19.7)	100 (100.0)
X^2	24.406			
P	0.000			

STI, sexually transmitted infection； HPV, human papillomavirus.

Supplementary Table 3 Comparison of the positive rates of detected STI pathogens between groups with or without HPV16/18 infection

**STIs**	**Total /N**	**HPV 16/18 positive N=204**	**HPV 16/18 Negative N=411**	**X^2**	**P**
C. trachomatis	75	25 (12.3%)	50 (12.2%)	0.001	1.000*
U. urealyticum	85	21 (10.3%)	64 (15.6%)	3.188	0.083
M. hominis	37	9 (4.4%)	28 (6.8%)	1.390	0.282
M. genitalium	9	5 (2.5%)	4 (1.0%)	2.065	0.166
U. parvum serovar 1	49	16 (7.8%)	33 (8.0%)	0.006	1.000*
U. parvum serovar 3	119	46 (22.5%)	73 (17.8%)	2.002	0.160
U. parvum serovar 6	132	45 (22.1%)	87 (21.2%)	0.064	0.835
U. parvum serovar 14	5	1 (0.5%)	4 (1.0%)	0.394	1.000*
N. gonorrhoeae	7	5 (2.5%)	2 (0.5%)	4.675	0.043
HSV-2	35	7 (3.4%)	28 (6.8%)	2.904	0.098*
Single infection	277	99 (48.5%)	178 (43.3%)	1.502	0.472
Multiple infection	126	39 (19.1%)	87 (21.2%)		

* Fisher's exact test. Multiple infections double counting.

HR-HPV, high-risk human papillomavirus; STIs, sexually transmitted infections; *C. trachomatis*, *Chlamydia Trachomatis*; *U. urealyticum*, *Ureaplasma urealyticum*; *M.*

hominis, *Mycoplasma hominis*; *M. genitalium*, *Mycoplasma genitalium*; *U. parvum*, *Ureaplasma parvum*; *N. gonorrhoeae*, *Neisseria Gonorrhoeae*; HSV-2, herpes simplex virus 2.
